# Supplementary material for: Low-Intensity Blue Light Exposure Reduces Melanopsin Expression in Intrinsically Photosensitive Retinal Ganglion Cells and Damages Mitochondria in Retinal Ganglion Cells in Wistar Rats
Source: Cells. 2023 Mar 26;12(7):1014. doi: 10.3390/cells12071014 (PMC10093228; doi:10.3390/cells12071014)
Supplement: Supplementary file 1 [file cells-12-01014-s001.zip › cells-2274784 - Table S1.pdf]

Table S1. Counts of TUNEL- positive nuclei in the outer nuclear layer of control rats and of rats exposed to blue light.

Section	Short-term exposure									Long -term exposure									Control exposure								
	Animal									Animal									Animal								
	**1**	**2**	**3**	**4**	**5**	**6**	**7**	**8**	**9**	**1**	**2**	**3**	**4**	**5**	**6**	**7**	**8**	**9**	**1**	**2**	**3**	**4**	**5**	**6**	**7**	**8**	**9**
**1**	1	0	2	1	0	2	1	1	1	9	5	4	4	6	6	3	2	4	0	0	0	0	1	0	0	0	0
**2**	1	1	1	2	1	0	2	0	0	4	4	6	5	5	6	4	4	3	1	0	0	0	0	1	0	0	0
**3**	0	0	0	0	0	4	0	2	1	6	7	2	8	4	4	8	6	8	0	1	0	0	0	0	0	1	0
**4**	1	2	3	1	2	1	0	1	0	7	3	7	7	6	3	6	3	7	0	0	0	1	0	0	0	0	0
**5**	2	3	0	0	0	0	1	1	0	6	8	4	6	8	6	4	7	6	0	0	1	0	0	0	0	0	0
**6**	2	1	1	2	3	0	0	0	4	3	4	6	9	4	5	6	9	6	0	0	0	1	0	0	0	0	0
**7**	1	1	0	0	0	3	1	3	0	6	5	5	2	6	5	8	3	7	1	1	0	0	0	0	0	0	0
**8**	0	0	1	1	1	0	3	0	1	5	8	7	6	7	3	9	8	4	0	0	1	0	0	1	0	0	0
**SUM**	8	8	8	7	7	10	8	8	7	**46**	**44**	**41**	**47**	**46**	**38**	**48**	**42**	**45**	2	2	2	2	1	2	0	1	0
**MEAN**	**7.888**									**44.11**									**1.33**								
**SD**	**0.927**									**3.21**									**0.86**								
