# Supplementary figures and images for: Low-Intensity Blue Light Exposure Reduces Melanopsin Expression in Intrinsically Photosensitive Retinal Ganglion Cells and Damages Mitochondria in Retinal Ganglion Cells in Wistar Rats
Source: Cells. 2023 Mar 26;12(7):1014. doi: 10.3390/cells12071014 (PMC10093228; doi:10.3390/cells12071014)

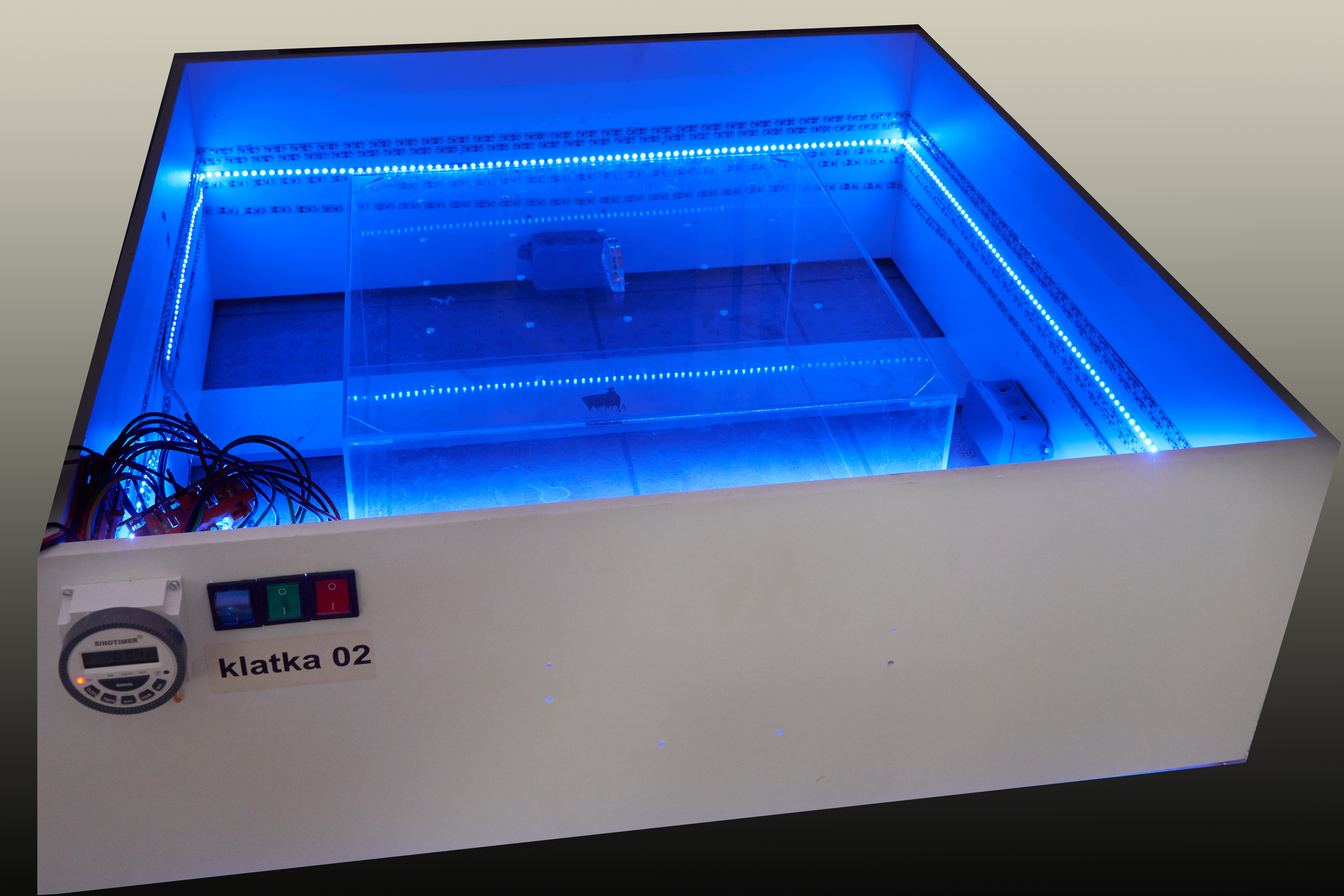

Supplement: Supplementary file 1 [file cells-12-01014-s001.zip › cells-2274784 - Figure S1.jpg]

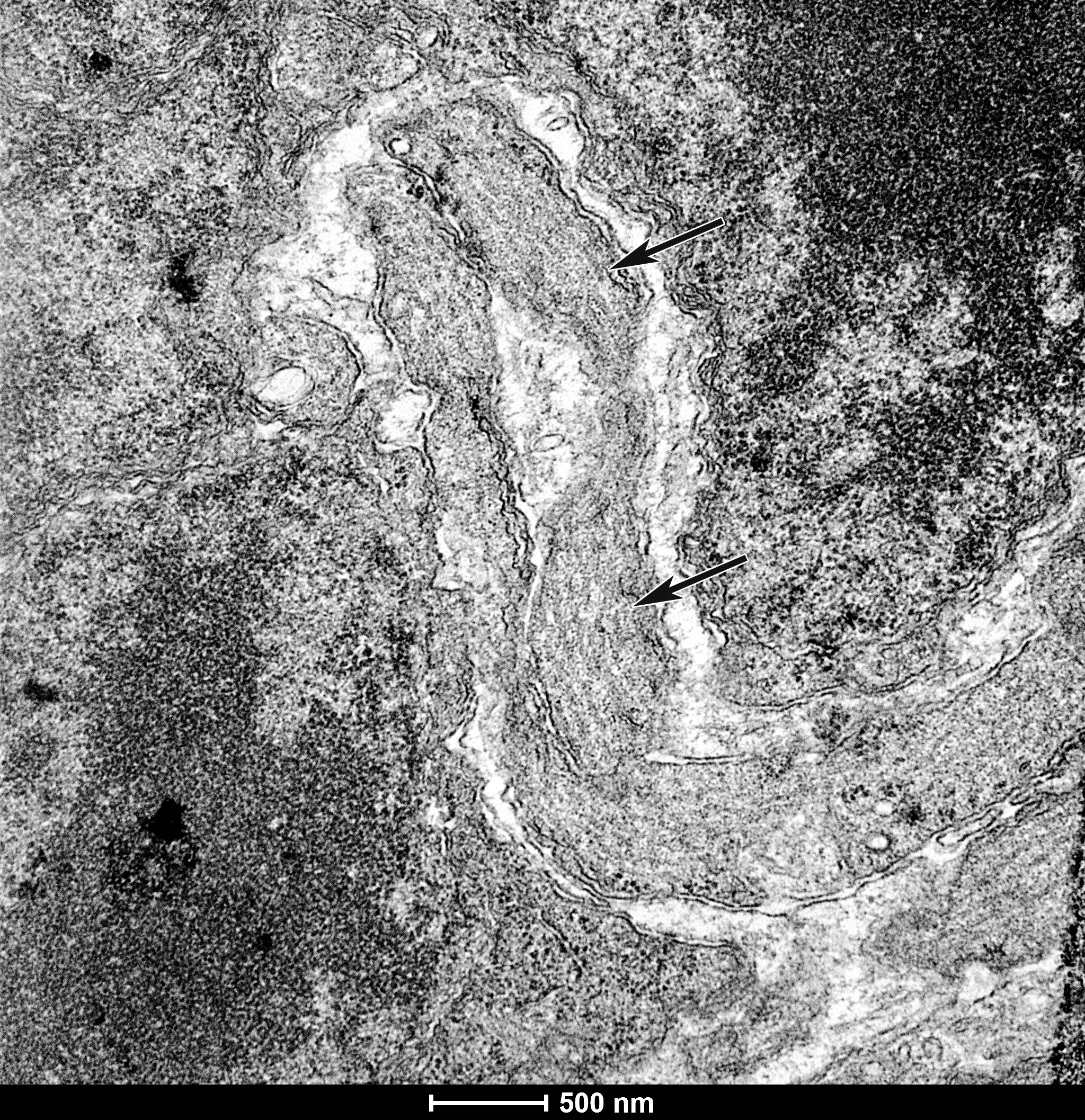

Supplement: Supplementary file 1 [file cells-12-01014-s001.zip › cells-2274784 - Figure S2.tif]
